# Targeting the Mitochondrial Permeability Transition Pore to Prevent Age-Associated Cell Damage and Neurodegeneration

**DOI:** 10.1155/2021/6626484

**Published:** 2021-01-28

**Authors:** Andrew C. Kent, Khairat Bahgat Youssef El Baradie, Mark W. Hamrick

**Affiliations:** ^1^Medical College of Georgia, Augusta University, Augusta, GA 30912, USA; ^2^University of Georgia, Athens, GA, USA; ^3^Faculty of Science, Tanta University, Tanta, Egypt

## Abstract

The aging process is associated with significant alterations in mitochondrial function. These changes in mitochondrial function are thought to involve increased production of reactive oxygen species (ROS), which over time contribute to cell death, senescence, tissue degeneration, and impaired tissue repair. The mitochondrial permeability transition pore (mPTP) is likely to play a critical role in these processes, as increased ROS activates mPTP opening, which further increases ROS production. Injury and inflammation are also thought to increase mPTP opening, and chronic, low-grade inflammation is a hallmark of aging. Nicotinamide adenine dinucleotide (NAD+) can suppress the frequency and duration of mPTP opening; however, NAD+ levels are known to decline with age, further stimulating mPTP opening and increasing ROS release. Research on neurodegenerative diseases, particularly on Parkinson's disease (PD) and Alzheimer's disease (AD), has uncovered significant findings regarding mPTP openings and aging. Parkinson's disease is associated with a reduction in mitochondrial complex I activity and increased oxidative damage of DNA, both of which are linked to mPTP opening and subsequent ROS release. Similarly, AD is associated with increased mPTP openings, as evidenced by amyloid-beta (A*β*) interaction with the pore regulator cyclophilin D (CypD). Targeted therapies that can reduce the frequency and duration of mPTP opening may therefore have the potential to prevent age-related declines in cell and tissue function in various systems including the central nervous system.

## 1. Introduction

The number of older adults is growing worldwide. As a result, the incidence of age-associated diseases including AD, osteoporosis, sarcopenia, and osteoarthritis is also increasing. This increase in age-related disorders has a significant, negative impact on the quality of life for patients and their families and also places a substantial burden on healthcare systems. A better understanding of the cellular and molecular mechanisms underlying aging is central to the successful development and clinical translation of novel therapies and prevention strategies. Recent work has demonstrated that changes in mPTP function may contribute directly to cellular dysfunction with aging [1–3]. These changes include increases in ROS production, induction of cellular senescence (particularly in aging stem cells), and activation of the inflammasome, the latter contributing directly to the chronic state of inflammation often referred to as “inflammaging” [1–3]. mPTP dysfunction has been cited as a key factor in neurodegenerative pathologies through its role in collapsing mitochondrial membrane potential, repressing mitochondrial respiratory function, releasing mitochondrial Ca^2+^ and cytochrome c, and enhancing ROS generation [4–7]. Thus, the mPTP has received increased attention as a potential therapeutic target.

The relationship between the mPTP and the generation of mitochondrial reactive oxygen species (mROS) has attracted significant interest within the context of aging and age-related tissue degeneration [8]. Recently, it was found that mROS can stimulate the opening of the mPTP, which can lead to further mROS production and release [9]. This positive feedback mechanism ultimately leads to an excessive amount of ROS accumulation. ROS accumulation in turn damages nuclear DNA, activates proapoptotic signaling pathways, and drives cellular aging [10–12]. On the other hand, ROS can in some cases activate protective pathways, decrease stress on the mitochondria, and increase lifespan [1, 11]. It is currently thought that the mPTP plays an important role in integrating the effects of mROS and hence may play a vital role in the aging process [8]. In this review, we discuss the various mechanisms inducing activation of the mPTP and the age-associated cell damage seen as a byproduct of mPTP activation. Furthermore, we discuss potential therapies that target the mPTP and may therefore inhibit the effects of aging and injury.

### 1.1. Structure and Formation of the mPTP

Various structural components of the mPTP are implicated in permeability transition (PT); however, the overall structure of the mPTP is still not completely understood. It was previously thought that the pore consisted of several components including a voltage-dependent ion channel (VDAC), an adenine nucleotide transporter (ANT), and a peripheral benzodiazepine receptor [13, 14]. These elements are described to perform specific roles: VDAC is associated with the benzodiazepine receptor and regulates the extramitochondrial transfer of cholesterol to the intermembrane space whereas ANT permits the inflow of phosphorylated and nonphosphorylated derivatives of adenine nucleotides [15]. Except for ANT, which is thought to act as a potential regulatory molecule, recent genetic experiments have ruled out the aforementioned elements as components of the mPTP [16]. Thus, we present here the most recent models regarding mPTP composition with the understanding that these may be revised in the near future.

Recent models of pore composition posit that the F_1_F_0_ (F)-ATP synthase is the main component of the pore and that the regulatory molecule CypD is a protein modulator of the mPTP [17]. In this model, the mPTP originates from a conformational change occurring on the F_1_F_0_ (F)-ATP synthase after Ca^2+^ binding, possibly by replacing Mg^2+^ at the catalytic site [18]. Whether the dimeric form or the monomeric form of F_1_F_0_ (F)-ATP synthase is necessary to increase PT is still of great debate [19, 20]. Nevertheless, F_1_F_0_ (F)-ATP synthase's status as a pore component is supported by genetic manipulation of F_1_F_0_ (F)-ATP synthase [20, 21], by electrophysiological measurements [20, 22–24], and by mutagenesis of specific residues of F_1_F_0_ (F)-ATP synthase [18, 25–27]. On the other hand, Walker and colleagues have proposed that the F_1_F_0_ (F)-ATP synthase is not an essential component of the pore [28, 29]. Their hypothesis is based on the observation that, even after ablating subunits b and OSCP of F_1_F_0_ (F)-ATP synthase, mitochondrial PT still occurred [29]. Matrix swelling was used to determine PT because long-lasting mPTP opening in vitro is followed by solute diffusion with matrix swelling [30].

Questions have, however, been raised regarding these findings. Bernardi [17] in particular noted the absence of replicates and calibration with pore-forming agents like alamethicin may complicate interpretation of the data. The effects on respiration following F_1_F_0_ (F)-ATP synthase knockout raise additional questions. Respiratory activity was dramatically decreased to between 10 and 20% of the rate observed in wild-type cells after F_1_F_0_ (F)-ATP synthase knockout [29]. The driving force in respiring mitochondria for Ca^2+^ accumulation is the inside-negative membrane potential generated by respiration [31, 32]. Furthermore, Ca^2+^ uptake is charge-compensated by increased H+ pumping by the respiratory chain [17]. Thus, it is important to note that the maximal rate of Ca^2+^ uptake is limited by the maximal rate of H+ pumping by the respiratory chain [33]. When extramitochondrial Ca^2+^ levels exceed 2 *μ*M, the latter becomes rate-limiting [34]. He et al. [28] used 10 *μ*M pulses of Ca^2+^ to induce PT; therefore, Ca^2+^ uptake by mitochondria lacking subunits c, b, and OSCP should have been significantly lower and not identical to wild-type mitochondria [17]. This raises questions about the Ca^2+^ retention capacity, a measurement used by He et al. [28] to determine mPTP opening. It is possible that respiratory inhibition due to absence of certain subunits may not be constant over time. Potential mechanisms may exist that restore the expression of F_1_F_0_ (F)-ATP synthase and by consequence the respiratory chain. When considering the above findings, F_1_F_0_ (F)-ATP synthase cannot necessarily be ruled out as a pore component.

**Figure 1:**
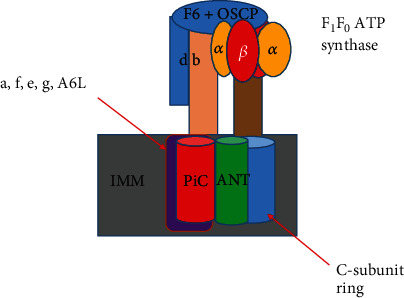
Prevailing model concerning the makeup of the mPTP as formed by the following potential components: mammalian F_1_F_0_ (F)-ATP synthase, Adenine nucleotide translocator (ANT), and mitochondrial phosphate carrier (PiC). The figure is redrawn and adapted based on reference [138]. Although ANT and PiC remain controversial potential components of the mPTP, they are shown as both red and green components overlaying the inner mitochondrial membrane (IMM). Similarly, although F_1_F_0_ (F)-ATP synthase is a controversial component, it is labeled as follows. Subunits of the F_0_ component labeled in purple include a, e, f, g, and A6L. F_1_ components include *α* and *β* subunits labeled in yellow and red, respectively. The C ring subunit is labeled in blue represented by a cylinder. The F_1_ peripheral stalk is composed of the subunits b, d, F6, and oligomycin sensitivity conferring protein (OSCP) labeled represented by a peach rectangle, a blue rectangle, and a blue circle, respectively. The mPTP is the point at which ROS, Ca^2+^, and other molecules can escape from the matrix of the mitochondria.

The most compelling experiments supporting F_1_F_0_ (F)-ATP synthase as a pore component focus on the mutagenesis of specific residues of F-ATP synthase. Specifically, it was found that matrix H+ leads to inhibition of mPTP and complete channel block at pH 6.5 [25, 35]. It was found that the mPTP block is mediated by reversible protonation of matrix-accessible His residues [35]. Recently, H112 of the OSCP subunit has been implicated as the unique His responsible for the PTP block by H+ [25]. Although these findings are intriguing with respect to mPTP activity, they serve a dual purpose in also supporting OSCP and by consequence F_1_F_0_ (F)-ATP synthase as potential components of the mPTP. Further controversial components include ANT, which may serve a regulatory role by binding CypD and reconstituting into proteoliposomes, producing Ca^2+^-activated pores similar to the mPTP [36, 37] and the mitochondrial phosphate carrier PiC [38, 39]. Thus, potential constituents of the mPTP include ANT, PiC, and F_1_F_0_ (F)-ATP synthase (Figure 1). Although its role is controversial, we emphasize the potential role of F_1_F_0_ (F)-ATP synthase in mitochondrial permeability.

F_1_F_0_ (F)-ATP synthase's various interactions with molecules such as CypD result in increased mitochondrial permeability. The specific subunits of F_1_F_0_ (F)-ATP synthase have been studied in relation to their interaction with regulatory molecules such as CypD. It is thought that mammalian F_1_F_0_ (F)-ATP synthase is a protein complex composed of the following: an F_1_ region composed of (*αβ*)3, *γ*, *δ*, and *ε* subunits, which protrudes in the matrix and synthesizes/hydrolyzes ATP; an F_0_ sector, formed from *a* subunit, the c8-ring, two membrane-inserted *α*-helices of b subunit, and supernumeraries subunits e, f, g, k, A6L, diabetes-associated protein in insulin-sensitive tissue (DAPIT) and 6.8 kDa proteolipid, which allows H+ flow across the IMM; the central stalk complex; and the peripheral stalk subcomplex composed of the following: oligomycin sensitivity conferral protein (OSCP), d, F6, and the extrinsic *α*-helices of A6L and b subunits (Figure 1) [40].

OSCP and CypD interact to promote the opening of the mPTP, and further, mPTP opening is increased with aging and oxidative stress [41–43]. Oxidative stress induces the translocation of the tumor suppressor p53 to the mitochondrial matrix where it interacts with CypD to aid in the formation of the mPTP [44]. Like the oxidative stress-induced formation of the mPTP, Ca^2+^ can also induce formation of the mPTP. It has been found that soluble matrix peptidylprolyl isomerase F cyclophilin D (PPIF) is involved in the Ca^2+^-induced opening of the mPTP [15]. The interaction between the aforementioned molecules, oxidative stress, and Ca^2+^ overloading can change significantly across the lifespan.

### 1.2. Role of the mPTP in Cellular Aging

A range of studies indicates that mPTP activation is altered with age in a variety of cell and tissue types. These include permeabilized myofibrils in humans [45], myocytes in rats [46], and osteocytes in mice [47]. It should be noted, before discussing the various effects of aging on mPTP activation, that Ca^2+^ is a well-established activator of the mPTP [48]. Specifically, concerning the permeabilized myofibrils in humans, Gouspillou et al. [28] found that Ca^2+^ retention and time to mPTP opening were significantly decreased in skeletal muscle of older active men [45]. Decreased Ca^2+^ retention is indicative of mPTP openings [45]. It was also found that the mPTP of older, active men maintains an increased sensitivity to Ca^2+^, further supporting the idea that increased mPTP activation is a byproduct of aging. These results are further reinforced by work showing that oxidative damage to Ca^2+^ transporters leads to Ca^2+^ leakage into the cytosol and subsequent mitochondrial matrix Ca^2+^ overloading, which then leads to activation of the mPTP [49, 50].

**Figure 2:**
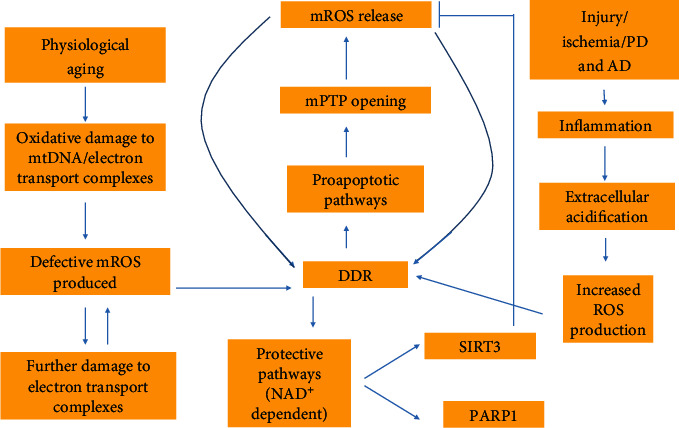
All of the following contribute to ROS production: physiological aging, injury, ischemia, PD, and AD. Injury, ischemia, PD, and AD do so by means of inducing inflammation. Extracellular acidification is a pathological effect of inflammation. A decrease in extracellular pH leads to increased ROS production within the cell which in turn instigates DDR. Aging results in oxidative damage to either mtDNA or electron transport complexes. This instigates defective mROS production. Upon ROS-induced ROS release, ROS can damage nuclear DNA, again inducing DDR. DDR results in proapoptotic pathways that induce mPTP opening and further mROS release. A positive feedback mechanism is initiated in which mPTP openings allow for mROS release which instigates DDR. Simultaneous to the proapoptotic mechanism is the NAD^+^-dependent protective pathways. SIRT3 in particular acts as an inhibitor to mROS release. It is important to note that these mechanisms are opposing and upon depletion of NAD^+^, the proapoptotic pathways dictate mROS release as the protective pathways are unable to perform their function.

Activation of the mPTP can also be seen as a product of increased ROS production. Notably, ROS production increases with age [51], and it is thought that ROS production is increased in complexes I and III with the inhibition of electron transport [52]. Oxidative damage to mtDNA and/or electron transport complexes is suggested to result in defective ROS-producing complexes. A cycle is established in which ROS produced by damaged mtDNA and/or electron transport complexes further damages electron transport complexes with age [51] (Figure 2). The increase in ROS production with age is noteworthy because increased mPTP activation is associated with elevated levels of ROS. This is based on the study conducted by Zorov and colleagues, who found that ROS accumulation within the mitochondria of cardiac myocytes leads to increased mitochondrial permeability transition and release of ROS from the mitochondria (ROS-induced ROS release) [53]. Thus, a clear relationship between age, elevated ROS levels, and increased mPTP openings is established. As will be discussed later, ROS released from the mitochondria can damage nuclear DNA and lead to proapoptotic signals which increase mPTP openings [54–56]. Due to the scope of this article, changes in the respiratory chain with aging will not be discussed further.

ROS-induced ROS release is observed during aging and after injury. Inflammation, a process typically associated with injury, induces extracellular acidification [57]. This acidification can in turn lead to increased ROS production within the cell [58]. Increased ROS production in the cell instigates mROS release from the matrix of the mitochondria [9], specifically by means of the mPTP [59]. Thus, inflammation can effectively alter the function of the pore by increasing PT. These effects are not, however, limited to inflammation. Ischemia is also known to decrease extracellular pH [60], in turn launching the same ROS stimulating pathway described above in which the release of mROS further stimulates the production of ROS leading to a positive feedback mechanism in which normal pore function is disrupted [8].

Intracellular pH, like extracellular pH, plays a role in the interaction between inflammation, ischemia, and mPTP activation. Kerr et al. [61] used 2-deoxy-d-[3H]glucose (2-DG) mitochondrial entrapment to show that recovery of Langendorff-perfused rat hearts from ischemia is accompanied by a reversal of the mitochondrial PT [61]. This connection hinges on pyruvate, which is suggested to inhibit the mPTP by decreasing intracellular pH. The beneficial effects of mPTP inhibition included recovery of left ventricular pressure [61]. When considering the results of their study, it is clear that mPTP function is altered in ischemic injury, specifically by means of increased permeability. Yet, it is this same alteration of increased permeability that further stimulates injury, as evidenced by the beneficial effects observed upon mPTP inhibition. The protective effects of mPTP inhibition are further evidenced by Na(+)-H(+) exchanger-1 (NHE-1) inhibition. NHE-1 inhibition in hearts subjected to ischemia/reperfusion using the same 2-DG mitochondrial entrapment method described above is associated with attenuation of mPTP opening [62]. The beneficial effects of mPTP attenuation also included recovery of left ventricular pressure [62]. A careful analysis of these studies shows that increased PT is observed with injury, inhibition of the mPTP can lead to a decrease in PT, and decreased PT can improve cardiac function.

ROS production within the cell leads to mPTP opening and subsequent mROS release. It is thought that the outer-membrane anion channel, VDAC, plays a role in allowing the release of ROS from the intramembranous space of the mitochondria [63]. The ROS that are released by VDAC include superoxide and H_2_O_2_, as they are both small enough (less than 1500 kDa) to pass through the channel [64]. Once released into the cytosol, ROS damages nuclear DNA [11] and triggers the DNA damage response (DDR). DDR induces both proapoptotic signaling in postmitotic pathways [12] and protective pathways [11] (Figure 2). Proapoptotic signals include p53, which targets the mitochondrial matrix, and p66Shc, which targets the intermembrane space. p66Shc induces apoptosis specifically by means of generating H_2_O_2_. H_2_O_2_ reacts with cytochrome c and induces oxidation of the mPTP leading to mitochondrial swelling and ultimately mPTP activation [54–56]. Thus, the increase in ROS production seen as a byproduct of aging initiates mPTP opening, but mPTP opening leads to further ROS production (H_2_O_2_) via proapoptotic signals. This positive feedback mechanism is a means by which continued opening of the mPTP leads to a destruction of the membrane potential, swelling, and rupture of the outer mitochondrial membrane. The mPTP exacerbates the effects of aging as the rupture of the outer mitochondrial membrane leads to the release of ROS, Ca^2+^, and other metabolites which can, in turn, induce oxidative damage to proteins, transporters, and nuclear DNA ultimately disrupting cellular homeostasis [9, 50].

**Figure 3:**
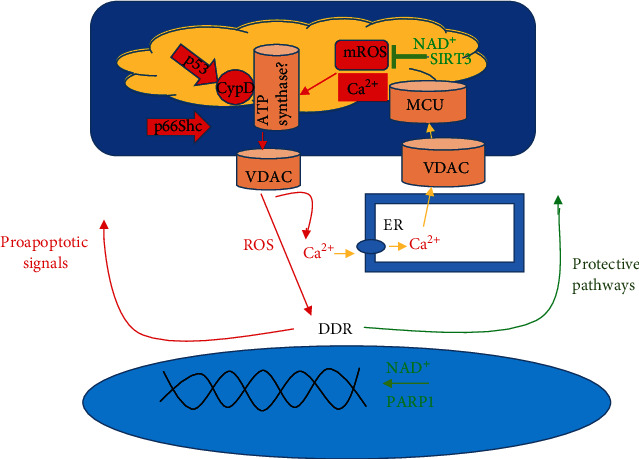
The components of the mPTP are of great controversy. However, despite this, CyPD and the controversial F_1_F_0_ (F)-ATP synthase are shown as pore constituents. VDAC, while not considered to be part of the mPTP, is thought to be how mROS, Ca^2+^, etc. are shuttled from the intermembrane space to the cytosol. mROS release through the mPTP leads to DNA and Ca^2+^ transporter damage. DNA damage induces DDR or DNA damage response. DDR subsequently induces both proapoptotic signals and protective pathways. Proapoptotic signals recruit p53 and p66Shc which act upon the mPTP (p53 specifically interacts with CypD, and p66Shc targets the intermembrane space generating ROS) to further induce mPTP openings. Oxidative damage to Ca^2+^ transporters can lead to calcium overloading and subsequent increased mPTP openings. MCU in particular can be affected by oxidative damage, leading to a disruption in mitochondrial Ca^2+^ levels. Protective pathways such as PARP1 aid in DNA repair, and SIRT3 inhibit mROS production. As further oxidative damage to DNA takes place, both protective pathways continue to utilize NAD^+^. NAD^+^ depletion can result, leading to an inactivation of protective pathways. In turn, the proapoptotic signals are left unchallenged and mPTP openings become more frequent.

The frequency of mPTP opening is further increased by Ca^2+^ overloading in the matrix [49, 50, 65] Ca^2+^ concentration within the mitochondria is driven by cytosolic Ca^2+^ levels and mediated by the Ca^2+^ uniporter MCU [66, 67]. It is known that aging disrupts Ca^2+^ homeostasis [68, 69] and interferes with the interaction between ER and mitochondria [70]. The disruption in Ca^2+^ homeostasis is thought to be a byproduct of oxidative damage to Ca^2+^ transporters which increases the leak of Ca^2+^ into the cytosol and subsequently increases Ca^2+^ overload of the mitochondria [71, 72] (Figure 3). Since oxidative damage to Ca^2+^ transporters is a byproduct of increased ROS levels, the continued opening of the mPTP would lead to further damage first initiated by cellular aging. In addition to damaged Ca^2+^ transporters, the direct transfer of calcium from the ER to the mitochondria increases Ca^2+^ overloading within the matrix [73]. To counter calcium overloading resulting from mPTP openings, MICU1, a subunit of MCU, limits calcium accumulation in the matrix as it maintains a threshold for calcium uptake [66, 74]. In aged cells, however, cytosolic free calcium often exceeds the MICU1 threshold for calcium uptake while the calcium threshold controlling mPTP activation is lower than the normal threshold [75]. This would indicate that more Ca^2+^-induced mPTP openings are to be observed in aged cells. Since ROS release can lead to oxidative damage of Ca^2+^ transporters and consequently Ca^2+^ overloading, increased mPTP sensitivity with age can be seen as a byproduct of both Ca^2+^ overloading and ROS release. Furthermore, mPTP opening can be seen as a key driver of the processes (oxidative damage to Ca^2+^ transporters, etc.) first initiated by aging.

### 1.3. Protective Pathways Involving PARP1 and SIRT3 Can Inhibit mPTP Opening

Although aging can increase ROS production, ROS do not always invoke damaging effects. This is because protective pathways exist to counter oxidative damage by ROS. Thus, due to the dual nature of ROS, which can have both protective and damaging effects, it is necessary to address the interplay between both to examine the overall effects of mPTP opening. Before the interplay can be discussed in regard to aging, it is necessary to examine the protective pathways stimulated by mROS that exist to maintain cellular homeostasis. One of the most important pathways begins with the intranuclear protein PARP1. When DNA is damaged through oxidative stress, PARP1 repairs DNA in a manner that is dependent on NAD^+^ [76]. Working in conjunction with PARP1 are the sirtuins, which are similarly classified as NAD-dependent deacetylases [77]. It is thought that SIRT3 plays a particularly important role in inhibiting ROS production and mPTP activation in stem cells [78]. Through metabolic reprogramming, SIRT3 is suggested to effectively increase efficient electron transport away from carbohydrate catabolism, resulting in reduced ROS production [79, 80]. Thus, because ROS production is decreased and mPTP activation is inhibited, the protective pathways above antagonize the mPTP from exacerbating the processes initiated by aging. With this in mind, it is clear that any downregulation of the protective pathways described above through depletion of NAD^+^ would lead to the domination of mPTP openings and subsequent positive feedback regarding the release and production of mROS.

As aging proceeds, NAD+ depletion, mPTP openings, and mROS production and release lead to DNA damage. The protective pathways noted above, as well as activation of nuclear factor erythroid 2-related factor 2 (Nrf2), are initiated to counter oxidative damage; however, the continued activation of these pathways leads to a depletion of NAD^+^. Since both the PARP1 and sirtuin protective pathways are dependent upon NAD^+^, they are no longer able to perform their function as an inhibitor of mPTP opening and subsequent mROS release and production [81, 82]. Ultimately, as aging progresses, oxidative damage to nuclear DNA results in the activation of protective pathways which in turn leads to depletion of NAD^+^. Without NAD^+^, the protective pathways involving PARP1 and SIRT3 are unable to perform their function as an mPTP opening inhibitor. As a consequence, the mPTP can effectively exacerbate processes initiated by aging.

SIRT3 serves a protective role to inhibit ROS production but it can also act to deacetylate CypD and inhibit mPTP opening [83]. Since SIRT3 is dependent upon NAD^+^ levels, CypD openings are therefore also dependent at least in part on NAD^+^ levels. Consequently, as NAD^+^ concentration declines as a byproduct of protection pathway activation, SIRT3 is unable to perform inhibition of CypD-induced mPTP opening [81, 83]. SIRT3's simultaneous effects on ROS production and CypD lead to an interplay between the two, which enables further mPTP openings. As discussed earlier, a decrease in SIRT3 activity leads to proapoptotic pathway activation through the ROS-induced DDR [12, 77]. In particular, p53 binds to CypD to form a complex triggering mPTP openings [44]. Thus, ROS production and CypD activation are connected via SIRT3 inhibition.

Pore openings are not limited to CypD's interaction with p53. Recently, a relationship was established between metformin, AMP kinase (AMPK), the peroxisome proliferator-activated receptor-*α* (PPAR*α*)/mitochondria pathway, and CypD in cardiomyocytes [84]. It is known that activation of AMPK protects the heart from myocardial infarction and heart failure [84]. Thus, because it was found that metformin activates AMPK, metformin can be a potential impetus in driving myocardial protection. Metformin abolished oxidative stress-induced physical interactions between PPAR*α* and cyclophilin D (CypD), and the abolishment of these interactions was associated with inhibition of mPTP formation [84]. Thus, the myocardial protective effects of metformin were found to converge at the mPTP.

### 1.4. Aging, Decreased Lifespan, and Neurodegenerative Diseases as Byproducts of mPTP Opening

mPTP openings become more frequent and longer in duration as a byproduct of increased ROS production with age and subsequent ROS-induced ROS release [51, 53]. mPTP openings lead to the release of ROS which in turn stimulates proapoptotic pathways leading to further openings [12, 54–56]. Due to the protective pathway dependence on NAD^+^, depletion of NAD^+^ leads to the inhibition of protective pathways leaving the counter effects of proapoptotic signals to proceed unchallenged [81, 83]. Thus, it is necessary to address the effects of mPTP opening in regard to the overall phenomenon of aging. It has long been held that ROS accumulation leads to oxidative stress and the subsequent observable phenomenon of aging [85]. Recently, ROS involvement in cellular senescence has received significant attention with regard to organismal aging. Cellular senescence is thought to be initiated by genomic damage which activates DDR and subsequent pathways leading to growth arrest [86]. The accumulation of senescent cells in organismal tissue is commensurate with advancing age, and senescent cells reduce stem and progenitor cell numbers leading to impaired capacity for tissue regeneration [87–89]. Considering that ROS play a vital role in cellular senescence and the mPTP plays a vital role in the release of ROS, it is therefore likely that the mPTP contributes to the progression of senescence.

Despite knowledge on the interaction between ROS and the mPTP, little work has been done with regard to the relation between the mPTP and cellular senescence. Hofer and colleagues investigated rat ventricular subsarcolemmal (SSM) and interfibrillar (IFM) mitochondrial susceptibility to Ca^2+^-induced mPTP openings with aging and calorie restriction [90]. They found that IFM exhibited an increased susceptibility towards mPTP openings during senescence. A decline in Ca^2+^ retention was observed with aging, particularly during senescence [90]. It is important to note that SSM did not exhibit these same results, and mPTP's association with senescence may be dependent on the tissue type. SSM aside, these results would suggest that the mPTP plays a role in the induction of cellular senescence and thus tissue aging, as evidenced by the decline in Ca^2+^ retention. As described above, genomic damage initiates the cellular pathway inducing senescence. Since it is known that ROS release through the mPTP is capable of inducing DDR, it is clear that a relation between mPTP opening, induction of cellular senescence, and cell and tissue aging exists.

Another proposed mechanism by which mPTP opening leads to cellular aging is through increased levels of autophagy. While autophagy is commonly thought to increase longevity due to its ability to clear damaged proteins and dysfunctional organelles, it can be detrimental at very high levels [91]. Elevated autophagy shortened lifespan in *C. elegans* lacking serum/glucocorticoid-regulated kinase-1 (sgk-1) because of increased mitochondrial permeability [91]. Furthermore, mice maintaining sgk-1 displayed lower levels of mitochondrial permeability, normal levels of autophagy, and normal lifespan. Based on these results, sgk-1 is suggested to modulate mPTP opening, which in turn mediates mitochondrial permeability, autophagy, and lifespan [91]. Since mitochondrial permeability is enhanced in the absence of sgk-1, it can be concluded that lifespan reduction as a byproduct of elevated autophagy is likely due to increased mPTP activity.

Research on neurodegenerative diseases, particularly on PD, has uncovered significant findings regarding mPTP openings and aging. PD is characterized by two phenomena including loss of dopaminergic neurons in the *substantia nigra* [92] and accumulation of highly insoluble fibrillar aggregates of the protein alpha-synuclein [93]. Recently, Ludtmann and colleagues [64] investigated the relationship between monomeric and oligomeric *α*-synuclein encoded by the gene SNCA and their subsequent effects on mPTP openings and cellular death. While *α*-synuclein in its monomeric form improves ATP synthase efficiency, upon protein aggregation and subsequent formation of the oligomeric form, a toxic gain of function is observed. Specifically, as it relates to the mPTP, the oligomers induce selective oxidation of the ATP synthase beta subunit resulting in an increased probability of mPTP opening. This finding is significant as induced pluripotent stem cell- (iPSC-) derived neurons bearing SNCA triplication generate *α*-synuclein aggregates that interact with ATP synthase and induce mPTP opening, leading to neuronal death [94].

PD is, however, not fully characterized by neuronal death alone. Loss of the antioxidant protein (protein-disulfide reductase) glutathione (GSH), a reduction in mitochondrial complex I activity, increased oxidative damage of DNA, and elevated free iron levels in the *substania nigra* have all been documented in patients suffering from PD [95, 96]. As mentioned earlier, ROS production increases with age, specifically in complexes I and III with the inhibition of electron transport [51, 52]. Furthermore, ROS accumulation within the mitochondria can lead to ROS-induced ROS release via the mPTP [53]. ROS released from the mitochondria can damage nuclear DNA and lead to proapoptotic signals which stimulate further mPTP openings [54–56]. Thus, the mPTP links two key processes associated with PD: a reduction in mitochondrial complex I activity leading to increased mitochondrial ROS, which in turn prompts mPTP openings and subsequent ROS release inducing increased DNA damage [51–56]. It is also important to note that neuroinflammation is observed in PD [64], and inflammation leads to extracellular acidification [33] which in turn leads to increased ROS production in the cell driving further mROS release from the matrix of the mitochondria via the mPTP [5, 34, 35]. The etiology of PD is complex and multifactorial involving environmental factors, genetic susceptibility, and aging that together promote disease progression [97]. The findings reviewed above suggest that the mPTP is also likely to have a potential role in the pathogenesis of PD.

Age-related dysfunctions of the mPTP extend to and are prevalent in age-related pathologies mediated by various factors such as inflammation. Inflammation is an early step in the pathogenesis of AD [98], and neuroinflammation is a process that occurs in PD [99]. As was noted earlier, extracellular acidification can increase ROS production, which leads to increased PT via the opening of the mPTP [59]. mPTP dysfunction may also be involved in the progression of AD. In its later stages, AD is characterized by massive amyloid-beta (A*β*) deposition in the parenchyma and the cerebrovascular walls [100, 101]. Recent findings show that mitochondrial damage in AD is linked to A*β* toxicity [102–105]. Some examples include decreased mitochondrial respiratory chain function [105, 106], increased mitochondrial ROS generation [105, 107], and changes in mitochondrial structure [108]. The interaction of A*β* species with certain regulators of the mPTP is likely responsible for the aforementioned damage. Specifically, the interaction of A*β* species with CypD and the upregulation of CypD expression were found to decrease the threshold of mPTP activation [109]. An AD mouse model overexpressing a mutant human form of amyloid precursor protein (mAPP) has also been shown to demonstrate increased CypD levels [109]. Thus, A*β* appears to be an important mediator connecting AD to the mPTP.

CypD is considered a crucial component for mitochondrial permeability transition pore (mPTP) formation [4, 110]. Du et al. found that mitochondrial function and learning/memory were significantly improved in CypD-deficient mice [109, 111]. These results suggest that pore formation is a necessary step in the pathogenesis of AD and that the ablation of CypD in mice gives lifelong protection against A*β*-induced mitochondrial and behavioral dysfunction [111]. Other studies have shown that A*β* oligomers induce a massive entry of Ca(2+) in neurons and promote mitochondrial Ca(2+) overload and mitochondrial PT [112]. This is significant because, as mentioned earlier, Ca^2+^ overload can lead to mPTP activation [49, 50]. Nonsteroidal anti-inflammatory drugs (NSAIDs), including salicylate and sulindac sulfide, were able to inhibit mitochondrial Ca^2+^ overload through mitochondrial depolarization. These studies highlight the role of mPTP dysfunction in neurodegenerative disease.

### 1.5. Potential Therapies to Mitigate mPTP Opening

Previous work suggests that mPTP opening plays a role in both injury and aging, thus targeted therapies to inhibit continued and frequent opening of the mPTP may serve to promote longevity and healthspan (Table 1). As discussed earlier, PD, AD, and other age-related disorders are thought to be byproducts of mPTP openings. Research targeting the mPTP whether directly or indirectly is divided into two areas. The first area involves therapeutics that require some form of interaction with CypD, and the second area involves therapeutics that require no interaction with CypD [113].

Of the therapies that inhibit CypD, cyclosporin A (CsA) has evoked great interest as it has shown cytoprotective properties in cellular models due to its ability to interfere with the interaction of CypD with the mPTP [114]. Specifically, CsA has been shown to block mitochondrial Ca^2+^ efflux and allow mitochondria to accumulate large amounts of Ca^2+^ [115]. The mechanism which is responsible for increased Ca^2+^ retention is indicative of mPTP closure [45]. This point is supported by Crompton and colleagues, who found that ability of mitochondria to retain Ca^2+^ in the presence of CsA was due to CsA inhibition of the mPTP [116]. CypD in particular was shown to be the target of CsA [117]. mPTP openings were studied in ischemic reperfusion injury in rat hearts to determine the efficacy of CsA with regard to cardioprotection. Cardioprotection was observed in a narrow range, between 0.2 and 0.4 *μ*M, as benefits were lost at concentrations above 0.4 *μ*M [118]. Despite these promising results, a recent clinical trial showed that CsA failed to improve clinical outcomes and prevent adverse left ventricular remodeling in patients with an acute anterior ST-segment elevation myocardial infarction (STEMI) [117]. This raises questions regarding the viability of targeting CypD to promote cardioprotection. It is possible that these seemingly conflicting studies on the cardioprotection offered by CsA could be explained by the means of drug administration. Since cardioprotection was observed in a narrow range in rat hearts, it is possible that the dosage administered in the clinical trial, 2.5 mg/kg body weight, was too low/high of a concentration [117].

Further research supporting CypD as a viable cardioprotective target was conducted by Parodi-Rullman et al. on induced myocardial infarction in rats [119]. They found that CypD inhibition exerts cardioprotective effects in reperfused but not in nonreperfused infarcted hearts of female rats, and the effects are observed only during acute postinfarction injury. CypD remains a viable target for age-related pathologies, although the timing and dosage of drug administration should be refined and optimized to demonstrate clear benefits for the patient. The CypD inhibitor, CsA derivative N-methyl-isoleucine-4-cyclosporin (NIM811), has been investigated as a therapeutic alternative to CsA alone. In a study conducted to determine the efficacy of NIM811 with regard to inhibiting the mPTP, it was found that both mitochondrial permeability transition onset and apoptosis were prevented when NIM811 was added to rat hepatocytes [114]. The potential of NIM811 for reducing mitochondrial permeability and improving cell survival has also been shown in animal models of spinal cord injury [120], traumatic brain injury [121], and hindlimb ischemia-reperfusion injury [122].

CypD-independent therapeutics have received attention (Table 1). Melatonin in particular has been studied as a potential inhibitor to the mPTP that does not require CypD interaction. Melatonin has been shown to inhibit mPTP activation as evidenced by reduced mitochondrial swelling and increased Ca^2+^ capacity [123]. This is further supported by Andrabi et. al who studied the effects of melatonin on mPTP openings in rat brain models [124]. The release of cytochrome c was used to assess pore opening, and rats treated with melatonin displayed a marked decrease in cytochrome c release [124]. These results would support the assertion that melatonin does indeed inhibit mPTP activation. Postmortem analyses of cerebrospinal fluid shows a marked decrease in melatonin concentration with age [125], which could in theory contribute to increased mPTP opening with aging. mPTP openings lead to swelling of the mitochondria, rupture of the outer mitochondrial membrane, and subsequent release of intermembranous proteins [126]. Melatonin supplementation may therefore represent one option to suppress mPTP opening in older adults who are likely to have relatively low endogenous levels of melatonin.

In addition to melatonin, other CypD-independent therapeutics include mitotargeted compounds (Table 1). Mitotargeted therapeutics acting in the absence of CypD interaction include electron scavengers, cinnamic anilides, N-phenylbenzamides, and isoxazoles. One small molecule that directly targets the mPTP is (E)-3-(4-fluoro-3-hydroxy-phenyl)-N-naphthalen-1-yl-acrylamide (compound 22), a cinnamic anilide that inhibits oxidative stress and chemical crosslinker-induced mPTP opening [127]. Other novel CypD-independent therapies exist, classified in the same cinnamic anilide series and performing similar functions as compound 22. One example is GNX-4728 which was administered in a mouse model of amyotrophic lateral sclerosis (ALS). GNX-4728 was found to slow disease progression, improve motor function, and extend lifespan by nearly twofold. Furthermore, Ca^2+^ retention was established, which is again indicative of mPTP closure [128]. Regarding N-phenylbenzamides, the most prominent therapeutic candidate is compound 4, (3-(benzyloxy)-5-chloro-N-(4-(piperidin-1-ylmethyl)phenyl)benzamide). Compound 4 induced a concentration-dependent increase in the calcium retention capacity (CRC) of permeabilized HeLa cells suggesting mPTP inhibition [129]. The isoxazole, compound 1, 5-(3-hydroxyphenyl)-N-(3,4,5-trimethoxyphenyl)isoxazole-3-carboxamide, produced similar results in an isolated mouse liver mitochondria model. Compound 1 was shown to inhibit mitochondrial swelling without interfering with the inner mitochondrial membrane potential [130].

Electron scavengers are mitotargeted therapeutics acting in the absence of CypD interaction. Some of the most studied drug therapies in this category include SS-31, XJB-5-131, MitoQ, EUK-8, and MitoTEMPO. SS-31 can increase cell survival and reduce intracellular ROS in neuronal N2A cells treated with t-butylhydroperoxide (tBHP) [131]. XJB-5-131 can improve postischemic recovery of aged hearts, reduce Ca^2+^-induced swelling in mitochondria, and reduce total mROS levels in cardiomyocytes [132]. It was also observed that XJB-5-131 improved motor skills and cognitive functions in rats with traumatic brain injury. These results are seen as a byproduct of decreased levels of mROS and subsequent prevention of cardiolipin oxidation [133]. Both MitoQ and EUK-8 employ the same electron-scavenging mechanisms as the therapies above. MitoQ's therapeutic effects were examined in rat cardiac ischemia-reperfusion injury and MitoQ decreased cell death and decreased mitochondrial damage [134]. EUK-8's effects were examined in presymptomatic heart/muscle-specific manganese-superoxide dismutase- (Mn-SOD-) deficient mice. It was observed that EUK-8 suppressed the progression of cardiac dysfunction and diminished ROS production and oxidative damage [135]. Again, while the above therapies do not interact directly with the mPTP, they do reduce ROS levels or production within the mitochondria, which leads to inhibition of mPTP opening.

MitoTEMPO has been investigated as a potential therapeutic in the treatment of AD. A recent study was performed in which A*β* toxicity, a hallmark of AD, was measured in primary cultured mouse neurons. Upon treatment with MitoTEMPO, it was found that A*β*-induced mitochondrial superoxide production and neuronal lipid oxidation were significantly decreased. Furthermore, a protective effect on mitochondrial bioenergetics was observed as evidenced by preserved mitochondrial membrane potential [136]. These results would indicate that MitoTEMPO has the potential to protect neuronal function in AD. While the previous therapies are in the developing stages, one therapy had been approved for treating acute ischemic stroke in Japan. Edaravone is a free radical scavenger that produces neuroprotective effects. The mechanism in which this is accomplished is similar to the other scavengers in that edaravone captures and reduces excessive ROS [137]. Similarly, as with the other scavengers, the therapy acts on the relationship between ROS and mPTP activation. Thus, as a byproduct of edaravone administration, a decrease in ROS is observed and a decrease in mPTP activation occurs.

## 2. Summary and Conclusions

Mitochondrial dysfunction is now thought to play a significant role in the tissue degeneration and loss of function that occurs in multiple organ systems with advanced age. A key factor in this process is the generation of reactive oxygen species in mitochondria of aged cells, which is in turn associated with cell death, senescence, and tissue damage. The mitochondrial permeability transition pore appears to play a significant role in ROS production with aging. For example, the continued opening of mPTP and release of mROS lead to DNA damage. Cytoprotective pathways are activated to counter oxidative damage; however, the continued activation of these pathways leads to a depletion of NAD+. Since both the PARP1 and sirtuin protective pathways are dependent on NAD+, they lose their ability to suppress mPTP opening and inhibit mROS release and production. These findings point to mPTP inhibition as a potential therapeutic target for age-related disorders. Mitotargeted compounds and small molecules such as NIM811 have, at least in animal models, demonstrated success in promoting cell survival in settings associated with significant cellular damage such as spinal cord injury, traumatic brain injury, and ischemic stroke. Despite this, the application of mPTP targeted drugs in a medical setting remains elusive. This is evidenced by CsA, which failed to improve clinical outcomes and prevent adverse left ventricular remodeling in patients with an acute myocardial infarction. The electron scavenger edaravone remains one of the only mPTP-targeted drugs approved for clinical use as a neuroprotective agent. Future studies should be directed at exploring more long-term use of these small molecules in older animals to determine their effects on the development and progression of chronic age-associated disorders of the brain, musculoskeletal, and cardiovascular systems.

## Figures and Tables

**Table 1:** Small molecules targeting the mPTP, directly or indirectly, and their potential impact on age-associated diseases.

Classification	Compound	Experimental model	Effect
CypD inhibition independent	Melatonin	Rat stroke model	Decreased neuron loss and reduced infarct volume [124]
CypD inhibition dependent	Cyclosporin A (CsA)	Ischemic reperfusion injury rat heart [118]; anoxia-induced injury in rat heart myocytes [139]	Cardioprotection [118]; reduced proportion of necrosis in rat heart myocytes [139]
	N-Methyl-isoleucine-4-cyclo-sporin (NIM811)	Isolated mitochondria in rat hepatocytes (TNF*α*-induced permeable transition onset)	Mitochondrial permeability transition onset and apoptosis prevented [114]
CypD inhibition independent	(E)-3-(4-Fluoro-3-hydroxy-phenyl)-N-naphthalen-1-yl-acrylamide (compound 22)	Rabbit model of acute myocardial infarction	Cardioprotective; reduced infarct size; inhibitor of mPTP openings [127]
	Edaravone (Radicut™)	Ischemia/reperfusion injury in rat brain	Neuroprotective; inhibited Ca^2+^- and H_2_O_2_-induced swelling in mitochondria; inhibited Ca^2+^ generation of ROS [140]
N-Phenylbenzamides (CypD inhibition independent)	Compound 4, (3-(benzyloxy)-5-chloro-N-(4-(piperidin-1-ylmethyl)phenyl)benzamide)	Ca^2+^ retention capacity (CRC) assay in HeLa cells	Induced a concentration-dependent increase in the CRC of permeabilized HeLa cells (indicative of mPTP inhibition) [129]
Isoxazoles (CypD inhibition independent)	Compound 1, 5-(3-hydroxyphenyl)-N-(3,4,5-trimethoxyphenyl)isoxazole-3-carboxamide	mPTP openings measured by CRC in isolated mouse liver mitochondria	Inhibitory activity against mitochondrial swelling; no interference on the inner mitochondrial membrane potential [130]
Cinnamic anilides (CypD inhibition independent)	GNX-4728	Mouse model of amyotrophic lateral sclerosis (ALS)	Slowed disease progression and significantly improved motor function; displayed a nearly 2-fold extension of lifespan; established mitochondrial calcium retention [128]
Electron scavengers/antioxidants (CypD inhibition independent)	SS-31	15-month-old male mice exposed to isoflurane [141]; antioxidant properties of SS peptides in neuronal N_2_A cells treated with t-butylhydroperoxide (tBHP) [131]	Enhances synaptic plasticity and provides protective effects on cognitive function [141]; reduced intracellular ROS and increased cell survival [131]
	XJB-5-131	Cardiolipin oxidation as a byproduct of experimental traumatic brain injury in rats [133]; cardiac function in aged rats [132]; muscle contractility of aged adult rats [142]	Inhibition of cardiolipin oxidation and subsequent improvement in motor skills and cognitive operations [133]; improved postischemic recovery of aged hearts, reduced Ca(2+)-induced swelling in the mitochondria, attenuated the H_2_O_2_-induced depolarization of the mitochondrial inner membrane as well as the total and mitochondrial ROS levels in cultured cardiomyocytes [132]; higher muscle contractility [142]
	MitoQ	Cardiac ischemia-reperfusion injury in rats	Decreased heart dysfunction, cell death, and mitochondrial damage after ischemia-reperfusion [134]
	EUK-8	Oxidative stress-sensitized harlequin (Hq) mutant mice and their wild-type (WT) counterparts [143]; presymptomatic heart/muscle-specific manganese-superoxide dismutase- (Mn-SOD-) deficient mice [135]	Improved left ventricular end-systolic dimensions and fractional shortening, prevented myocardial oxidant stress, attenuated necrotic and apoptotic cell death, and attenuated cardiac hypertrophy and fibrosis in both Hq and WT [143]; suppressed the progression of cardiac dysfunction and diminished ROS production and oxidative damage [135]
	MitoTEMPO	Amyloid-beta toxicity in primary cultured mouse neurons	Neuronal lipid oxidation was significantly suppressed; demonstrated protective effects on mitochondrial bioenergetics evidenced by preserved mitochondrial membrane potential and cytochrome c oxidase activity as well as ATP production [136]
